# Long-Term Effectiveness and Tolerability of Intranasal Esketamine in Treatment-Resistant Depression: A Real-World, Single-arm Study of Over 100 sessions

**DOI:** 10.1192/j.eurpsy.2026.10598

**Published:** 2026-07-31

**Authors:** N. Ayad, S. Makhoul

**Affiliations:** Reem Neuroscience Center, Abu Dhabi, United Arab Emirates

## Abstract

**Introduction:**

Treatment-resistant depression (TRD) poses a significant clinical challenge, with limited evidence guiding long-term pharmacological strategies. Esketamine, a glutamatergic modulator, has demonstrated short-term efficacy in TRD, but data on its extended use in real-world settings remains scarce

**Objectives:**

This study aimed to evaluate the long-term effectiveness and tolerability of intranasal esketamine in adults with TRD over more than 100 treatment sessions.

**Methods:**

We conducted a retrospective, single-arm pre-post study of 20 patients with TRD at a private psychiatric clinic in the United Arab Emirates. All participants received ≥100 sessions of intranasal esketamine alongside oral antidepressants. Depression and anxiety symptoms were assessed using the Patient Health Questionnaire-9 (PHQ-9) and Generalized Anxiety Disorder-7 (GAD-7) scales. Tolerability was monitored through blood pressure, sedation, dissociation, urinary symptoms, and psychiatric side effects.

**Results:**

After an average of 129 esketamine sessions (mean duration 2.5 years), PHQ-9 and GAD-7 scores significantly decreased (p<0.001). 85% of patients improved in depressive severity, with 25% achieving remission. 65% improved in anxiety severity, and 20% reached remission. Esketamine was generally well-tolerated; side effects were mild and transient, with no serious adverse events. No clinical or demographic factors significantly predicted response.

**Conclusions:**

Intranasal esketamine demonstrated sustained effectiveness and acceptable tolerability in a real-world TRD cohort with extensive psychiatric comorbidity. These findings support its long-term use in complex clinical populations and underscore the need for further prospective, multi-site studies.

**Disclosure of Interest:**

None Declared

